# Development of Reporting Guidelines for Animal Health Surveillance—AHSURED

**DOI:** 10.3389/fvets.2019.00426

**Published:** 2019-11-27

**Authors:** Arianna Comin, John Grewar, Gerdien van Schaik, Heinzpeter Schwermer, Julie Paré, Farouk El Allaki, Julian A. Drewe, Ana Carolina Lopes Antunes, Leah Estberg, Michael Horan, Francisco F. Calvo-Artavia, Abdurrahman Hassan Jibril, Marta Martínez-Avilés, Yves Van der Stede, Sotiria-Eleni Antoniou, Ann Lindberg

**Affiliations:** ^1^Department of Disease Control and Epidemiology, National Veterinary Institute, Uppsala, Sweden; ^2^South African Equine Health and Protocols NPC, Cape Town, South Africa; ^3^GD Animal Health, Deventer, Netherlands; ^4^Department of Animal Health, Federal Food Safety and Veterinary Office, Berne, Switzerland; ^5^Section of Terrestrial Animal Health Epidemiology and Surveillance, Canadian Food Inspection Agency, Saint-Hyacinthe, QC, Canada; ^6^Veterinary Epidemiology, Economics and Public Health Group, Royal Veterinary College, London, United Kingdom; ^7^Division for Diagnostics & Scientific Advice – Epidemiology, Technical University of Denmark, Lyngby, Denmark; ^8^United States Department of Agriculture, Center for Epidemiology and Animal Health, Fort Collins, CO, United States; ^9^SAT Division, Department of Agriculture, Food and the Marine, Celbridge, Ireland; ^10^Unit of Animal Health, Danish Veterinary and Food Administration, Glostrup, Denmark; ^11^Department of Veterinary and Animal Science, University of Copenhagen, Copenhagen, Denmark; ^12^Center for Animal Health Research, National Institute for Agricultural and Food Research and Technology, Madrid, Spain; ^13^Unit of Animal and Plant Health, Department of Risk Assessment and Scientific Assistance, European Food Safety Authority, Parma, Italy

**Keywords:** animal health surveillance, output-based standards, reporting guidelines, harmonization, expert elicitation

## Abstract

With the current trend in animal health surveillance toward risk-based designs and a gradual transition to output-based standards, greater flexibility in surveillance design is both required and allowed. However, the increase in flexibility requires more transparency regarding surveillance, its activities, design and implementation. Such transparency allows stakeholders, trade partners, decision-makers and risk assessors to accurately interpret the validity of the surveillance outcomes. This paper presents the first version of the Animal Health Surveillance Reporting Guidelines (AHSURED) and the process by which they have been developed. The goal of AHSURED was to produce a set of reporting guidelines that supports communication of surveillance activities in the form of narrative descriptions. Reporting guidelines come from the field of evidence-based medicine and their aim is to improve consistency and quality of information reported in scientific journals. They usually consist of a checklist of items to be reported, a description/definition of each item, and an explanation and elaboration document. Examples of well-reported items are frequently provided. Additionally, it is common to make available a website where the guidelines are documented and maintained. This first version of the AHSURED guidelines consists of a checklist of 40 items organized in 11 sections (i.e., surveillance system building blocks), which is available as a wiki at https://github.com/SVA-SE/AHSURED/wiki. The choice of a wiki format will allow for further inputs from surveillance experts who were not involved in the earlier stages of development. This will promote an up-to-date refined guideline document.

## Introduction

Surveillance of disease has an essential role in protecting the health and welfare of animals and humans. While, human health surveillance commonly relies on notifiable disease reporting and analysis of secondary data, animal health surveillance (AHS) places a stronger emphasis on collecting primary data via active sampling of animal populations. For example, such active surveillance will support the objective to fulfill trade requirements and ensure food safety (1). A review of the surveillance systems currently present in some European countries (2) and an investigation of the availability of surveillance information (3) showed that design and achievements of AHS are generally not well-documented in Europe, especially for non-notifiable diseases. There seems to be a lack of detail, consistency, transparency, and open access in both private and public sectors. Also, there is a limited use of output-based surveillance standards, which prescribe what the surveillance must achieve rather than how surveillance activities must be carried out (4). Output-based standards have been endorsed over recent years to provide more flexibility in surveillance planning and thus allow for more efficient surveillance systems. However, they require transparent and consistent sharing of information about surveillance design and achievements in order to enable assessments of equivalence.

During 2012–2015, the EU project RISKSUR (http://www.fp7-risksur.eu/) developed a series of decision support tools for the design of cost-effective risk-based AHS systems. The need for systematic documentation of design decisions was considered during the development of one of the tools: the design framework (5) (https://survtools.org/wiki/surveillance-design-framework/doku.php). The conceptual idea was that by moving toward output-based standards for surveillance and allowing greater flexibility in surveillance design, there will be an increased need for transparency about design features as well as how the activities are actually implemented. However, although the RISKSUR design tool is comprehensive, there is still a need for guidance on (i) what information is truly critical for assessing the quality of surveillance evidence and (ii) how to report the necessary information in a useful manner to decision makers and stakeholders. This challenge was partly addressed within the SANTERO project (http://santero.fp7-risksur.eu/), where the first steps toward producing a set of reporting guidelines to facilitate consistent and credible reporting of surveillance activities and their outcomes were taken. The work was subsequently followed up in the HOTLINE project (https://www.thehotlineproject.org/), funded by the European Food Safety Authority (EFSA), which aimed to facilitate harmonized assessment and reporting of disease occurrence information (6).

The overall goal of AHSURED is to produce reporting guidelines that support communication of surveillance activities in the form of narrative descriptions. This should not be confused with reporting of notifiable diseases according to certain procedures defined by a competent authority or international body like the World Organization for Animal Health (OIE).

The concept of reporting guidelines evolved from the field of evidence-based medicine and serves to improve consistency and quality of information reported in scientific journals. A few reporting guidelines directly relevant to the veterinary field are available, such as REFLECT (https://meridian.cvm.iastate.edu/reflect) and STROBE-Vet (https://meridian.cvm.iastate.edu/strobe). These recommendations target randomized controlled trials and observational studies reported in veterinary scientific literature. They are based on guidelines already developed for studies in humans (CONSORT and STROBE). Several more are available for studies in the medical field. For an overview, see https://www.equator-network.org/. However, none of these is directly applicable to AHS activities. This is possibly due to the limited use of active surveillance in human health. It can also be related to the different perspective in the need for reporting guidelines. Currently available guidelines aim at enhancing the consistent reporting of scientific studies in peer reviewed journals, and therefore have an academic/editorial journal perspective. In contrast, reporting of disease surveillance is usually carried out by governmental bodies and have a more policy-oriented perspective.

The aim of this paper is to present the first version of the Animal Health Surveillance Reporting Guidelines (AHSURED) and the process by which they have been developed. The adoption of AHSURED will promote a more consistent approach to communication of AHS activities and their outputs, for the benefit of stakeholders, trade partners, decision-makers and risk assessors.

## Materials and Methods

**Figure 1:**
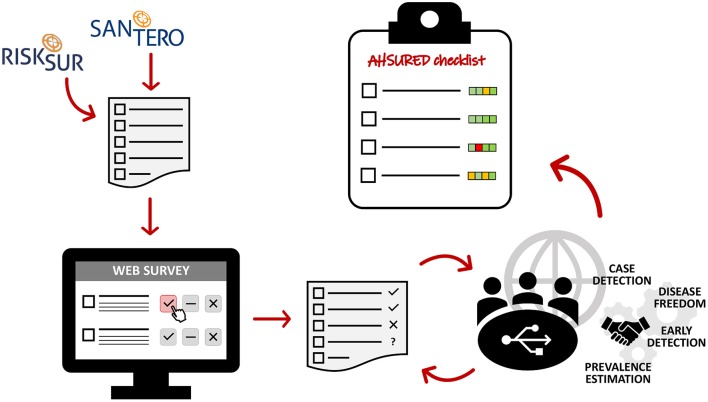
Overview of the development process of the animal health surveillance reporting guidelines (AHSURED).

Reporting guidelines usually consist of (i) a checklist of items to be reported; (ii) a description/definition of each item, (iii) an explanation and elaboration document (including examples of well reported items; and, optionally, (iv) a website where the guidelines are maintained and made available. As part of the process of having the guidelines endorsed by journal editors, it is common to publish the guidelines as well as a description of the process by which they have been developed. Thus, the current manuscript outlines the development process that led to the AHSURED guidelines. An overview of the process is provided in Figure 1.

### Provisional Checklist

Building upon the outcomes of the aforementioned projects (RISKSUR and SANTERO), a list of items that describe the building blocks of surveillance systems was initially identified. These represent elements that should be thought of and from which decisions are made during the design of a surveillance system. According to the definitions proposed by Hoinville et al. (7), a *surveillance system* was defined as a collection of various *surveillance components* which are all aimed at “describing the health-hazard occurrence and contributing to the planning, implementation, and evaluation of risk-mitigation actions.”

Mirroring the structure of the RISKSUR design tool, the identified descriptive items were grouped into building block sections. That could be either at the surveillance system level, e.g., the context in which the surveillance system specifically operates, or at surveillance component level, e.g., the target population of a specific surveillance component. The items to be included in each section evolved as part of the development process and are described in the results.

The RISKSUR design tool does not provide recommendations on how to present outcomes of surveillance activities. Therefore, four items were added under a “Results” section: (a) *Number of epidemiological units investigated (per stratum)*, (b) *Test results (per stratum)*, (c) *Surveillance outcomes (objective dependent)*, and (d) *Findings in relation to historical knowledge, or trend*. Item (c) means an assessment of the outcome of surveillance in relation to its objective. An example might be, for prevalence estimation, prevalence with an accompanying confidence interval. Furthermore, two sections with only one item each, titled “Interpretation,” providing the conclusions about the status of the population, and “References,” were added. The provisional checklist included 55 surveillance items organized in 15 sections and is available in Supplementary Material 1.

### Survey Among Surveillance Experts

The provisional list of surveillance items was subsequently presented to international AHS experts, who were identified through the distribution list of the third International Conference on Animal Health Surveillance (ICAHS, held in New Zealand in 2017) and contacted by email. In total, 621 invitations were sent out; of these, 115 were rejected by the mailing system and 506 were delivered.

The experts were consulted through a web-based survey and asked to indicate which items should be considered critical, optional or irrelevant to report when describing animal health surveillance designs and outputs. A critical item was defined as a piece of information that is deemed very important to report in order to understand and interpret surveillance outputs. An optional item is an additional piece of information that might enhance the interpretation of surveillance results if reported, but that will not hinder such interpretation if not reported. Items deemed irrelevant usually do not pertain to a specific surveillance objective. For example, items related to timeliness of detection might be unnecessary to report when demonstrating disease freedom.

The survey included 55 questions about the proposed surveillance items, 15 note fields to document any comment and/or suggestion (e.g., insertion or deletion of surveillance items) within each section, and five additional questions describing the background of the respondent. One last question asked whether the respondent was willing to further participate in the development of the guidelines. The outline of the survey can be found in Supplementary Material 2.

The survey was online between 31 October and 30 November 2018, and experts could provide their answers either as individuals or as collective groups of colleagues.

### Refinement and Consolidation

**Figure 2:**
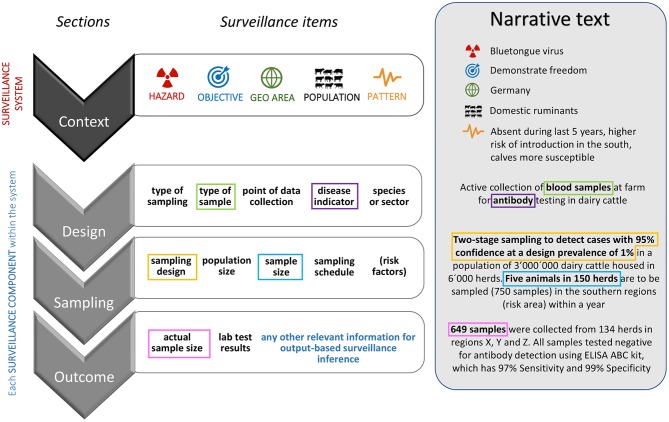
Schematic representation of some items describing a surveillance system (within white boxes) grouped by sections (gray shapes). The example in the shaded box refers to a hypothetical surveillance system to demonstrate freedom from Bluetongue virus and illustrates how each item could be conveyed within a narrative text reporting surveillance activities and outcomes. Colored squares identify the correspondence between some surveillance items and the relative text.

Starting from the provisional checklist and taking into account the insights gathered by the survey, the AHS reporting guidelines were finalized through consensus-oriented consultations limited to the group of international experts who had expressed willingness to contribute further to the process (further referred to as the core group of experts). These consultations were conducted through four thematic webinars, where the descriptive items were contrasted against example texts describing surveillance for different objectives (i.e., demonstrate freedom, early warning, detect cases, and estimate prevalence) and disease situations (i.e., absent, sporadically present, and endemic). A fictional example of the relation between the items of a checklist for surveillance reporting guidelines and a narrative text aiming at reporting surveillance activities and their outcomes is provided in Figure 2.

During each consultation, the checklist was iteratively revised in the light of a new specific surveillance objective. The emphasis of the discussion was on those items where <70% of the respondents of the survey agreed on the relevance of the item, i.e., if it was critical, optional or irrelevant to report. Flexibility was provided in the process to add or modify checklist items as necessary. The items' descriptions were carefully revised, and a new objective-specific assessment of their relevance was carried out by the core group of experts. The same applied to any new item suggested by the respondents to the survey. The four thematic webinars were held between 5 February and 1 April 2019.

## Results

At the end of the survey period, 33 web surveys were completed by 30 individuals and 3 groups of 5–10 co-workers. The respondents were geographically distributed across several parts of the world, although the majority worked in Europe (Table 1). Most of the respondents had more than 10 years of experience in animal health surveillance and mainly worked for State Authorities. The competence of the respondents was broad, ranging from design, implementation and evaluation of surveillance systems, to the analysis of surveillance data and risk assessment and risk management (Table 1).

**Table 1:** Background of the respondents to the online survey.

	**Individuals**	**Groups**	**Percentage of total**
Total number of respondents	30	3	
***From what areas of the world do you mostly have work experience?***			
Africa	5	0	15%
Asia	2	0	6%
Europe	18	2	61%
Oceania	1	0	3%
North America	3	1	12%
South America	1	0	3%
***What sector do you currently work for?***			
Public health	0	0	0%
Ministry/government	14	3	52%
Academia	8	0	24%
Private company	3	0	9%
Other	1	0	3%
***What is your current involvement in animal health surveillance?***			
***(multiple choice)***			
Design of surveillance activities	18	3	64%
Evaluation of surveillance performance	16	2	55%
Reporting surveillance results at national/international level	15	3	55%
Risk assessment	12	1	39%
Risk management	7	1	24%
Implementation of surveillance activities	16	3	58%
Secondary use of data (e.g., for research purposes)	16	2	55%
Other	0	0	0%
***How long have you been working with animal health surveillance?***			
Less than 5 years	2	0	6%
Between 5 and 10 years	7	1	24%
More than 10 years	21	2	70%

At least 70% of survey respondents agreed on the relevance of 40 of the 55 checklist items, which were all deemed to be critical to report. In particular: *Hazard under surveillance*; *Criteria for identification of suspicions;* and *Target unit level (unit of interest)* were judged critical by 97% of the respondents. In contrast: *Enhancements in place; When/how often are samples transferred; Who has performed the analyses; References;* and *Any other testing protocol details* scored almost 50:50 between critical and optional. Only 15 items were considered irrelevant by at least one respondent. Among these, *Institution involved and financing* was the item with the highest proportion of votes as irrelevant: 9% of the respondents. Details about the number of respondents that judged each item of the provisional list critical, optional or irrelevant can be found in Supplementary Material 3.

During the thematic webinars, 10–15 international surveillance experts evaluated the various items in the light of four surveillance objectives. These were: demonstrate freedom, early warning, detect cases, and estimate prevalence. They then re-assessed the relevance of those with inconclusive results from the survey through an iterative process of active discussion and (re)voting, until simple majority was obtained. Outcomes from the discussions included the improvement of item descriptions, the addition of one item that was suggested from the survey (i.e., *Identification of surveillance components*), deletion/grouping of some items deemed redundant, and combination of some sections. This iterative process led to a consolidated checklist consisting of 40 items organized in 11 sections, which is summarized in Table 2. The full AHSURED checklist with the detailed item descriptions is available as a wiki at https://github.com/SVA-SE/AHSURED/wiki. The choice of a wiki format was motivated by the fact that the collegial discourse leading up to the inclusion of certain items does not end with this manuscript and refinements and improvements of the guidelines are both expected and welcomed.

**Table 2:** Consolidated checklist^*^ of the surveillance reporting guidelines (A detailed description of each item is provided in the actual AHSURED guidelines, available at https://github.com/SVA-SE/AHSURED/wiki).

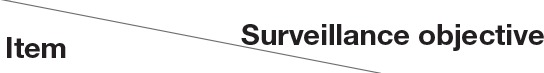	**Disease freedom**	**Case detection**	**Prevalence estimation**	**Early detection**
**1. Background**				
1.1. Hazard	Critical	Critical	Critical	Critical
1.2 Geographical area	Critical	Critical	Critical	Critical
1.3. Susceptible population	Critical	Critical	Critical	Critical
1.4. Historical situation	Critical	Critical	Optional	Optional
1.5. Surveillance purpose	Critical	Critical	Critical	Critical
1.6. Surveillance objective	Critical	Critical	Critical	Critical
1.7. Risk characteristics (level, aspect of risk)	Critical	Critical	Optional	Critical
1.8. Legal requirements and actions taken as a result of findings	Critical	Critical	Critical	Optional
1.9. Institutions involved and financing	Optional	Optional	Optional	Optional
1.10. Identification of surveillance components	Critical	Critical	Critical	Critical
1.11. Population strata not covered	Critical	Critical	Critical	Optional
**2. Surveillance component description**				
2.1. Data collection point	Critical	Critical	Critical	Critical
2.2. Surveillance approach	Critical	Critical	Critical	Critical
2.3. Type of hazard indicator	Critical	Critical	Critical	Critical
2.4. Type of material collected	Critical	Critical	Critical	Critical
2.5. Case definition	Critical	Critical	Critical	Critical
**3. Target population**				
3.1. Target criteria	Critical	Critical	Critical	Critical
3.2. Population coverage	Critical	Critical	Critical	Critical
**4. Enhancements**				
4.1. Enhancements	Optional	Optional	Optional	Optional
**5. Testing protocol**				
5.1. Pooling	Critical	Critical	Critical	Critical
5.2. Screening/first test	Critical	Critical	Critical	Critical
5.3. Confirmatory/second test	Critical	Critical	Critical	Critical
5.4. Accuracy of the testing protocol	Critical	Critical	Critical	Critical
**6. Study design**				
6.1. Sampling design	Critical	Critical	Critical	Critical
6.2. Number of target units in the target population	Critical	Critical	Critical	Critical
**7. Sampling strategy**				
7.1. Sampling at the primary sampling unit level	Critical	Critical	Critical	Critical
7.2. Sampling at the secondary sampling unit level	Critical	Critical	Critical	Critical
7.3. Sample allocation at the primary and secondary levels	Critical	Critical	Critical	Critical
7.4. Risk-based allocation	Critical	Critical	Critical	Critical
7.5. Sample size	Critical	Critical	Critical	Critical
7.6. Sample collection timeline	Critical	Critical	Critical	Critical
7.7. Who collects the samples	Optional	Critical	Optional	Optional
**8. Timeliness**				
8.1. Time from sampling to report	Irrelevant	Optional	Optional	Optional
8.2. Time from confirmation to action	Irrelevant	Optional	Irrelevant	Irrelevant
**9. Results**				
9.1. Number of epidemiological units investigated (per stratum)	Critical	Critical	Critical	Critical
9.2. Test results (per stratum)	Critical	Critical	Critical	Critical
9.3. Surveillance outcomes (objective dependent)	Critical	Critical	Critical	Critical
9.4. Findings in relation to historical knowledge, trend	Critical	Optional	Critical	Optional
**10. Interpretation**				
10.1. Surveillance interpretation	Critical	Critical	Critical	Critical
**11. References**				
11.1. References	Critical	Critical	Critical	Critical

* *Please note that the consolidated checklist is shorter than the one initially identified. Therefore, some items mentioned in the text are not present in this table, as they have been removed or modified during the refinement process. The initial provisional checklist of 55 surveillance items can be found in Supplementary Material 1*.

## Discussion

The development of animal health surveillance reporting guidelines was initiated in response to the increasing need for more transparent and systematic documentation of surveillance activities and their outputs (2). The development process involved the inputs from international AHS experts, identified through the distribution list of the third ICAHS conference. While, the mailing list included more than 500 experts, the number of respondents was low, suggesting the outcome of the questionnaire may not be representative of the opinion of the entire AHS community. In addition, although the respondents were geographically distributed across all habited continents and most of them had extensive experience in the field, the gathered view mainly reflects the AHS practices in industrialized countries, primarily Europe. This could possibly overlook the reality in developing and in-transition countries, where community-based management of disease suspicions and intersectoral collaborations play a stronger role (8). Nevertheless, the possibly constrained perspectives introduced by the non-representative sample of experts involved in the development process is mitigated by publishing the reporting guidelines as a wiki. In this way, the aim is to gather further inputs from surveillance experts who were not reached in the earlier stages, and to maintain the guidelines as a living document. In fact, the currently presented guidelines are to be considered only the first version of a document that will be hopefully expanded to account for additional surveillance strategies, like for instance participatory approaches (9).

The survey as the first step of the development highlighted that some items were prone to ambiguous interpretation of their relevance, as they scored almost 50:50 between critical and optional. This could be due to an unclear description of such items or, more likely, to a real duality of their relevance. For instance, *Findings in relation to historical knowledge* could be either critical or optional to report, depending on whether surveillance activities aim at documenting disease freedom or at detecting cases of disease, respectively. To disentangle ambiguities in views, a thematic approach was successfully adopted in the second step of the development by consensus-oriented consultations. Each thematic webinar focused on one of the four main objectives of surveillance and relative disease situations (i.e., absent, sporadically present, and endemic), thus allowing a deeper and more specific assessment of the relevance of each surveillance item.

The collection of information on AHS activities is often driven by requirements from national and international reporting bodies. Such requirements often have a strong influence on how AHS is developed and implemented (4). However, there is little harmonization in this respect, which hampers the ability to assess the equivalence of information and to benchmark surveillance activity (2, 10). Notably, there is a lack of metadata standards for AHS information, which is desirable when making these data not only publicly available but also useful and unambiguously interpreted. AHSURED will not solve this issue but may inform such standards since the AHSURED guidelines can be seen as a form of metadata definition, albeit more free in their format.

Unlike existing tools promoting structured ways to design or evaluate AHS [e.g., RISKSUR design and EVA tools (5, 11), SERVAL (12), SurF (13)], AHSURED does not involve any assessment of surveillance performances, but rather aims at documenting how surveillance activities were designed and carried out. The focus of AHSURED is really on communication, through the systematic description of how the output of surveillance have been generated. In that way, the reader is informed about aspects that influence the weight that can be put to the evidence in question.

The indication of whether an item on the checklist is critical, optional or irrelevant to report for a particular surveillance objective is not prescriptive but rather a suggestion on how important that piece of information is considered to be for understanding and interpreting the outputs of surveillance. Of course, any surveillance item can be reported, regardless of its objective-specific relevance. The adoption and ongoing refinement of AHSURED will improve a concise, transparent and consistent documentation and communication of surveillance activities and their outputs. This is crucial for the benefit of stakeholders, trade partners, decision-makers and risk assessors.

## Data Availability Statement

The datasets generated for this study are available on request to the corresponding author.

## Author Contributions

ALi and AC conceived and designed the study and wrote the first draft of the manuscript. AC implemented the web survey and summarized the results. ALi moderated the webinars. JG, GS, HS, JP, FE, JD, ALo, LE, MH, FC-A, AJ, MM-A, YV, and S-EA contributed to guidelines' refinement and consolidation. AC set up the wiki and all authors approved its content. All authors contributed to manuscript revision, read, and approved the submitted version.

### Conflict of Interest

The authors declare that the research was conducted in the absence of any commercial or financial relationships that could be construed as a potential conflict of interest. The reviewer JS declared a past collaboration with one of the authors FC-A.

## Supplementary Material

Supplementary Material 1 Provisional checklist of surveillance items.

Supplementary Material 2 Outline of the web survey.

Supplementary Material 3 Results of the web survey.
