# Supplementary material for: Development of Reporting Guidelines for Animal Health Surveillance—AHSURED
Source: Front Vet Sci. 2019 Nov 27;6:426. doi: 10.3389/fvets.2019.00426 (PMC6890601; doi:10.3389/fvets.2019.00426)
Supplement: Supplementary Material 1 — Provisional checklist of surveillance items. [file Data_Sheet_1.PDF]

Provisional checklist of surveillance items

This was the checklist that was initially identified as first step in the development of the AHSURED guidelines and further refined through a web-based survey and consensus-oriented consultations with Animal Health Surveillance experts.

The following is a list of items that describe the various **building blocks** of surveillance systems. They have been broken down into sections (belonging to either surveillance system or surveillance component level), namely:

1. Surveillance system – context
2. Surveillance component – component's characteristics
3. Surveillance component – target population
4. Surveillance component – disease suspicion
5. Surveillance component – enhancements
6. Surveillance component – testing protocol
7. Surveillance component – study design
8. Surveillance component – sampling strategy
9. Surveillance component – data generation process
10. Surveillance component – transfer means
11. Surveillance component – data translation process
12. Surveillance component – epidemiological analyses
13. Surveillance component – results
14. Surveillance system – interpretation
15. Surveillance system – references

Those items are intended to be part of a **checklist** within the surveillance reporting guidelines.

Colour coding:

Blue headings = optional information

Orange headings = information important mainly for surveillance aimed at early detection

Item | **Description/recommendations**

1 CONTEXT — Describe the context in which the surveillance system specifically operates

1.1 Hazard under surveillance

Hazard targeted by surveillance (a disease or another health-threat).

The surveillance activity may be multi-hazard. This is defined here as surveillance activities designed to target multiple hazards at the same time (parallel design), or the secondary use of data/samples collected as part of a surveillance activity designed for one specific hazard (mother component) to investigate the presence of additional hazards (child components). Should be mentioned if the surveillance of the hazard in question is part of a multi-hazard design.

1.2 Geographical area

The geographical area, which the surveillance is designed to provide evidence about.

1.3 Susceptible population

The specific animal population susceptible to the hazard in the geographical area of interest.

1.4 Historical evolution

Describe the historical status of the hazard in the area and population in question.

1.5 Surveillance objective

The objective of surveillance is a key characteristic which determines the most appropriate approach for the surveillance. See picture for clarification.

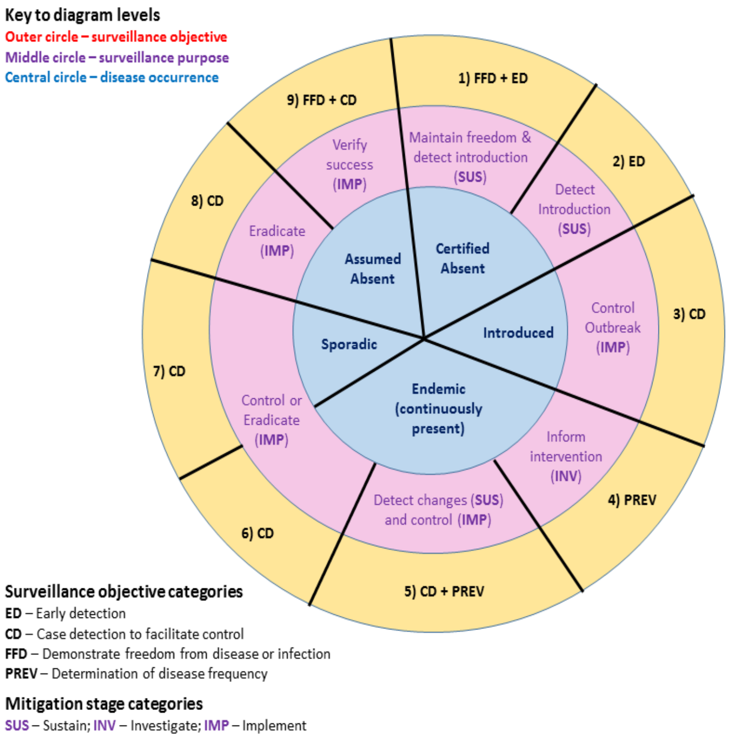

Prevalence estimation: This objective is appropriate if the target hazard is (thought to be) present in the territory to be covered, and an assessment of the prevalence and or changes in prevalence over time is needed.

Case detection: Case detection refers to the search for any animals affected, clinically or not, by the hazard under surveillance. This objective is appropriate if the hazard is present, regardless of whether the hazard occurrence is endemic, epidemic or sporadic, and action will be taken to control disease whenever it is detected.

Early detection: This objective is appropriate for a situation where the disease is currently absent in the country or region. It may be applied to surveillance for an unknown disease or a known disease considered to pose a non-negligible risk of incursion or emergence. It may also apply to the early stages of an outbreak.

Disease freedom: This objective is appropriate if the target hazard is thought to be absent in the territory to be covered and the aim is to demonstrate disease freedom for trade or non-trade purposes (e.g. improve public health, to decide when to stop an eradication programme and to eliminate production losses and control costs due to endemic disease). This may apply to diseases which a) have been historically absent, b) have been eradicated and official freedom has been confirmed or c) are thought to be eradicated, but official freedom has not yet been confirmed.

Surveillance systems may be used to provide information to address several surveillance objectives, but there is usually one that can be considered as primary. If other (secondary) surveillance objectives are relevant, they can be described for the components in question.

**1.6 Surveillance purpose**	How is the information collected in this surveillance system being used to inform policy decision (e.g. to eradicate or manage the occurrence of disease or inform trade)? See middle circle in the picture above for clarifications.
**1.7 Risk characteristics (level, aspect of risk)**	Risk characteristics associated with the hazard distribution and the susceptible population. Risk can be non-uniformly distributed at various level, such as population (particularly geographical and temporal risk factors), herd or animal. Risk characteristics should be described (whether they can be associated with a higher risk of hazard introduction, infection, detection, and/ or consequences).
**1.8 Legal requirements, actions taken as a result of findings**	Describe the legal support underlying the surveillance, and whether it is a requirement or not to carry out surveillance. Describe also if there are any legal obligations to report, or other requirements (may be associated with quality assurance schemes, declarations for trade etc).<br>Describe what actions are taken in case of a positive result, if any.
**1.9 Institutions involved, financing.**	Mention institutions involved in coordination, field work, laboratory work, financing. Describe the basis for financing.

**2 COMPONENT'S CHARACTERISTICS - To be described for each surveillance component separately**	
**2.1 Surveillance component objective**	Describe if different from main surveillance objective listed at 1.5<br>A surveillance system may for instance aim at demonstrating freedom (main objective) but one of its components (e.g. passive clinical surveillance) may aim at early detection (component-specific objective)
**2.2 Target species**	Describe if different from 1.3
**2.3 Target sector**	Describe if different from 1.3
**2.4 Geographical area**	Describe if different from 1.2
**2.5 Data collection point**	Where the units of interest can be reached, and therefore where samples will be collected.<br>This could be, e.g., at the source (farm, wild life habitat, etc), abattoir, coordination centre, artificial insemination centre, rendering plants, diagnostic laboratory, markets etc
**2.6 Study type**	Describe the type of study (study types in RISKSUR framework listed below);<br>(For passive surveillance components, please refer to sections 4 and 5).<br>• Survey<br>• Continuous data collection<br>• Sentinel surveillance<br>• Participatory surveillance<br>• Indicator-based surveillance<br>• Syndromic surveillance
**2.7 Type of disease indicator**	What outcome is measured for the unit of interest? E.g. is the surveillance aimed at antibody detection, pathogen detection, gross pathology, pathology diagnostic (microscopic) or indirect indicators etc?
**2.8 Type of sample collected**	What is the type of material collected? Examples are clinical reports, blood/serum/plasma, ear notches, tissue (biopsy), tissue (post mortem), milk, semen, urine, feces/ fecal swabs, other swab (not fecal), meat juice, environmental samples or feed.

3 TARGET POPULATION	Describe the target population of a specific surveillance component, which may be a subset of the whole population targeted by the surveillance system.
**3.1 Sectors missed**	Consider if there are other sectors/strata from the total susceptible population (defined in the surveillance scenario) that are not being covered by this particular component.
**3.2 Target criteria**	Describe any selection criteria used to choose this particular target population (if the target population for this component is not the entire susceptible population defined for the surveillance system). For example; logistic/convenience; higher probability of infection; higher probability of showing clinical signs; feasibility of detection (the diagnostic tests available can only be used in animals above a certain age, or non-vaccinated animals); or higher severity of consequences in case of infection.
**3.3 Percentage covered**	The percentage of the total susceptible population (defined in the surveillance scenario) covered by the target population defined for this specific component. This could be, for example, the percentage of the cattle population that is dairy for a component focused on dairy cows.

4 DISEASE SUSPICION	Describe how a suspected case of the hazard of interest is defined and reported to the relevant authorities. This is <u>relevant to passive surveillance components</u>, where the collection of surveillance data is observer-initiated
**4.1 Criteria for identification of suspicions**	Outline the definition or criteria used to identify a suspect case.
**4.2 Obligations on suspicions**	Describe requirements in place for reporting of a <u>suspect</u> case.
**4.3 Notification procedures**	Describe the procedures for reporting a suspect case, i.e. the steps involved and the methods employed, including how the notification is sent to the authorities.
**4.4 Actions upon suspicions**	Describe the procedures following the reporting of a suspect case to the authorities, for example at what stage would/could restrictions are applied to premises, or follow up investigations carried out.

5 ENHANCEMENTS	Describe any enhancements in place to encourage participation in surveillance and control activities, such as awareness campaigns and monetary or other incentives to reporting. This is <u>relevant to enhanced-passive surveillance components</u>, where the collection of surveillance data is observer-initiated after incentives
**5.1 Enhancements**	Examples are:<br>• payments or other financial rewards for notifications<br>• training to increase awareness and recognition of clinical signs<br>• awareness campaigns to improve recognition of disease and awareness of reporting obligations/procedures.<br>• payment of financial compensation for the losses the farmer may incur after a confirmation of notifiable disease<br>• provision of alternative routes of reporting such as a phone hotline or notification by SMS<br>• payment of the testing cost or some other form of mutual agreement such as farmer receiving advice in return<br>• mandatory/legal obligation to carry out diagnosis of exclusion

6 TESTING PROTOCOL	Describe how units have been tested to obtain information about the hazard
**6.1 Pooling**	If pooling has been done, please describe how and where (field, lab etc).
**6.2 Screening/first test**	Describe for the screening test (or only test), any thresholds for considering an animal as positive.
**6.3 Confirmatory/ second test**	For confirmatory tests (in cases where screening is used), or the second test (when using parallel tests), describe the test to be used, and any thresholds for confirming animals as positive.
**6.4 Any other testing protocol**	Any further details needed to make sure the entire process is well described.

7 STUDY DESIGN	Describe how the study population was selected
**7.1 Selection of units: census or sampling**	Describe if a census has been conducted, or if sampling has been applied. Describe the sampling frame used to select the units of interest.
**7.2 Target unit level (unit of interest)**	Describe the target unit, i.e. the level of the population for which conclusions are to be drawn (for instance animal or herds).
**7.3 Sampling unit - individual or group**	Describe the sampling units, i.e. the units that have actually been sampled (individuals; multiple group sample (collective/pooled samples which represent multiple animals, but not the entire target unit); one sample per group (collective/pooled samples which represent the entire group referred to in your target unit).
**7.4 Sampling design**	Describe the sampling design, e.g. one- or two-stage.
**7.5 Number of units in the target population**	Describe what is known about the size of the target population.
**7.6 Sensitivity of the testing protocol**	Describe what is known about the sensitivity of the testing protocol, incl. if tests are applied in series or parallel.
**7.7 Specificity of the testing protocol**	Describe what is known about the specificity of the testing protocol, incl. if tests are applied in series or parallel.

8 SAMPLING STRATEGY	Describe how units have been planned to be sampled
**8.1 Sampling at the primary sampling unit (PSU) level**	Information used to calculate sample size at the PS level, e.g. the number of PSU in the population (see section 7 also), design prevalence, desired confidence, desired power, sensitivity, specificity.
**8.2 Sampling at the secondary sampling unit (SSU) level**	Information used to calculate sample size at the SS level, e.g. the number of SSU in the population (see section 7 also), design prevalence, desired confidence, desired power, sensitivity, specificity.
**8.3 Selection criteria within the population**	See 3.6
**8.4 Risk-based allocation**	Describe the factors defining any risk strata. For each risk stratum defined, the following information should be reported:<br>a) risk characteristics: higher probability of infection, detection, consequences etc (revisit section 1.7)<br>b) percentage of the population that the stratum constitutes<br>d) relative risks

8.5 Sample size

The following details should be recorded:

a) sample size calculated at PSU level

b) sample size calculated at SSU level

c) planned distribution of samples over the year

8.6 Sample allocation at the primary and secondary levels

Describe the strategy for allocating samples.

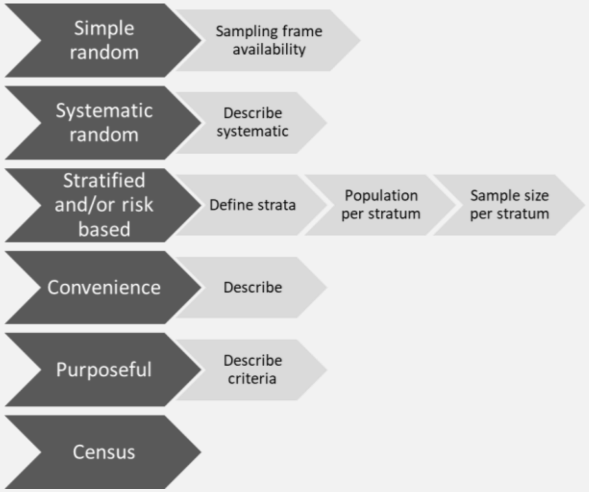

8.7 Sample collection timeline

Describe how the sampling has been distributed across the study period (sampling plan).

9 DATA GENERATION PROCESS

Describe the specific process of collecting the samples (or any other information) from the source

9.1 Who collects the samples?

Who are the agents who collect samples/information?

Examples of agents who may collect samples are:

- non-specialised actors (farmers, public, hunters, etc);
- technicians
- veterinarians.

9.2 When/how often are samples collected?

For surveillance where timeliness is important (early detection, case finding), describe how often samples have been collected across the study period.

10 TRANSFER MEANS

Describe how data/samples are transferred from the point of collection to the point of analyses

10.1 When/how often are samples transferred

For surveillance where timeliness is important (early detection, case finding), describe how often samples have been transferred across the study period.

11 DATA TRANSLATION PROCESS	Describe how the raw data (biological samples, health indicators, observations etc) is translated into surveillance information.
**11.1 Who has performed the analyses?**	Who has been responsible for the analysis of the raw data/samples, official laboratory status, accreditation etc
**11.2 When/how often are samples analysed**	For surveillance where timeliness is important (early detection, case finding), describe how often samples have been analysed across the study period. This could for example be: as soon as received (immediate/real-time); according to a fixed schedule eg weekly, monthly; or, in batches after a certain number of samples is reached.

12 EPIDEMIOLOGICAL ANALYSES	Once samples are analysed by the laboratories or interpreted by personnel, it is expected that the results of those analyses will be reviewed by epidemiologists and other involved in the study design for this surveillance component.
**12.1 When/how often is data analysed**	For surveillance where timeliness is important (early detection, case finding), describe how often data have been looked upon, analysed and interpreted during the study period.

13 RESULTS	Report the outcome of the surveillance activities for the described component.
**13.1 Number of epidemiological units investigated (per stratum)**	Report the number of epidemiological units investigated, by relevant strata and overall.
**13.2 Test results (per stratum)**	Report the outcome of the testing, by relevant strata and overall. Report sampling efforts results spatially and temporally.
**13.3 Surveillance outcomes (objective dependent)**	Provide an assessment of the outcome of surveillance objective in relation to its objective.<br>For prevalence estimation, this means prevalence with an accompanying confidence interval.<br>For case finding, the outcome of interest is incidence or the detection fraction.<br>For freedom studies, the outcome is probability of freedom, with accompanying design prevalence and confidence level (should be reported at several confidence levels/design prevalences).<br>For surveillance aimed at early detection, where no cases have been found, report intensity and relation to risk-based sampling activities.
**13.4 Findings in relation to historical knowledge, trend**	Provide a narrative and/or graphical description of the evolution of surveillance outcomes over time. If a trend analyses is carried out, describe method.

14 INTERPRETATION	
**14.1 Surveillance interpretation**	Summarise what evidence the surveillance activities provide about the status of the population, taking into account issues about coverage, representativeness/bias as well as generalisability. If relevant, describe any future plans to continue activities.

15 REFERENCES	
**15.1 References**	Provide references to support, e.g. methods and historical evidence.
