# Supplementary material for: Development of Reporting Guidelines for Animal Health Surveillance—AHSURED
Source: Front Vet Sci. 2019 Nov 27;6:426. doi: 10.3389/fvets.2019.00426 (PMC6890601; doi:10.3389/fvets.2019.00426)
Supplement: Supplementary Material 2 — Outline of the web survey. [file Data_Sheet_2.pdf]

AIM OF THE SURVEY

This survey aims at gathering insights on what is the **critical information** to provide when **reporting animal health surveillance outcomes** (not only for notifiable diseases), to further develop animal health surveillance reporting guidelines.

The rationale behind it is that by moving towards more output-based standards for surveillance, and allowing greater flexibility in surveillance design, there will be an increased need for transparency about design features as well as how the activities are actually implemented.
A comprehensive and structured list of items to consider when designing surveillance was earlier identified by the EU project RISKSUR (https://www.fp7-risksur.eu/); however, there is still a need for guidance on what information is truly critical for assessing the quality of surveillance evidence, and also on how to report it to decision makers and stakeholders. This task has been started within the SANTERO project (http://santero.fp7-risksur.eu/) and is now continued as part of the EFSA-funded project HOTLINE. The objective is to produce a set of reporting guidelines to facilitate consistent and credible reporting of surveillance activities and their outcomes.
The idea of reporting guidelines comes from the field of evidence-based medicine and serves to improve consistency and quality of information reported in scientific journals. A few reporting guidelines directly relevant to the veterinary field are available, such as REFLECT (http://www.reflect-statement.org/) and STROBE-Vet (https://strobevet-statement.org/). These statements target randomized controlled trials and observational studies reported in veterinary scientific literature and are based on guidelines already developed for studies in humans (CONSORT and STROBE). Several more are available for studies in the medical field (for an overview, see www.equator-network.org). However, none of these are directly applicable to surveillance activities.

The surveillance reporting guidelines developed so far can be found here: AHSURED_guidelines.pdf

STRUCTURE OF THE SURVEY

In this survey you will be first provided with some background information and terminology. Then, you will be asked to revise the surveillance reporting guidelines developed so far by answering to some questions.

The survey will take approximately **15-20 min** and can be answered individually or as a group. In the latter case, one person shall fill the questionnaire on behalf of the group.

The link to the survey that you received by email is **personal**, and will allow you to save your work and pick it up later, if needed. Please observe that whoever uses that link (e.g. if you forward the email to a peer) can override your answers.

Background information 1/2

This survey aims at gathering insights on what is the critical information to provide when reporting animal health surveillance outcomes.

This work builds on previous EU projects (RISKSUR and SANTERO), which, among other tasks, identified and described the building blocks of animal health surveillance.
The present survey will rely on the terminology adopted in the aforementioned projects, and in particular:

SURVEILLANCE: The systematic, continuous or repeated, measurement, collection, collation, analysis, interpretation and timely dissemination of animal health and welfare related data from defined populations. These data are then used to describe health hazard occurrence and to contribute to the planning, implementation, and evaluation of risk mitigation actions.

Surveillance information is gathered through surveillance systems.

SURVEILLANCE SYSTEM: A range of surveillance components (and the associated organisational structures) used to investigate the occurrence of a single hazard in a specified population.

SURVEILLANCE COMPONENT: A single surveillance activity (defined by the source of data and the methods used for its collection) used to investigate the occurrence of one or more hazards in a specified population.

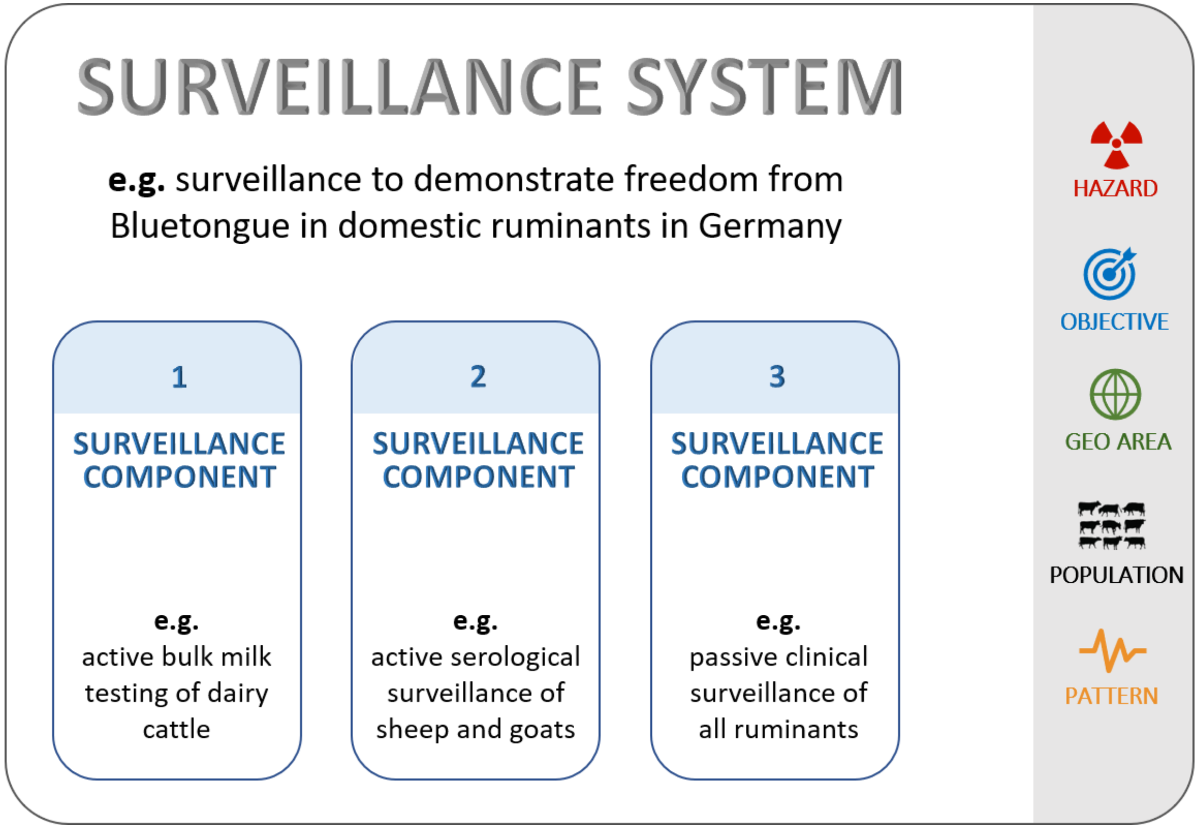

BACKGROUND INFORMATION 2/2

Understanding the process that led to a surveillance outcome is fundamental to correctly interpret the results. In fact, a surveillance outcome (e.g. 2 positive samples out of 100 tested) provides reliable information on health hazard occurrence only when put into a context. This can be done by describing the design/architecture of the surveillance activities (components) within the specific epidemiological situation (context) where they operate.

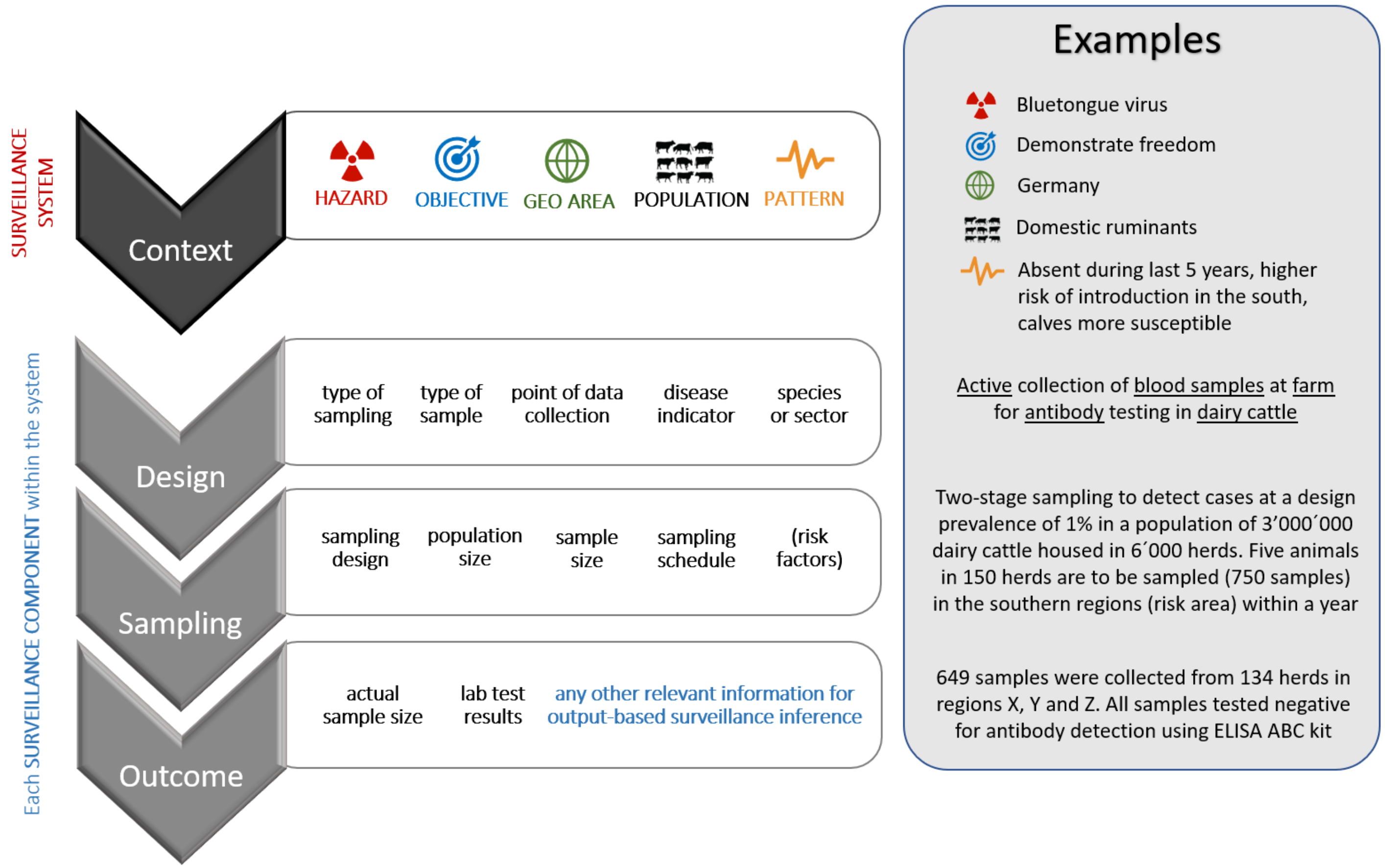

In this survey you will be presented with a list of items that describe the various building blocks of surveillance systems. For each of them, you will be asked to indicate whether you believe that it is critical, optional or irrelevant to correctly interpret surveillance outcomes.

ANIMAL HEALTH SURVEILLANCE REPORTING GUIDELINES

In this survey you will be presented with a list of items that describe the various building blocks of surveillance systems. They have been broken down into sections (belonging to either surveillance system or surveillance component level), namely:

- Surveillance system – context
- Surveillance component – component’s characteristics
- Surveillance component – target population
- Surveillance component – disease suspicion
- Surveillance component – enhancements
- Surveillance component – testing protocol
- Surveillance component – study design
- Surveillance component – sampling strategy
- Surveillance component – data generation process
- Surveillance component – transfer means
- Surveillance component – data translation process
- Surveillance component – epidemiological analyses
- Surveillance component – results
- Surveillance system – interpretation
- Surveillance system – references

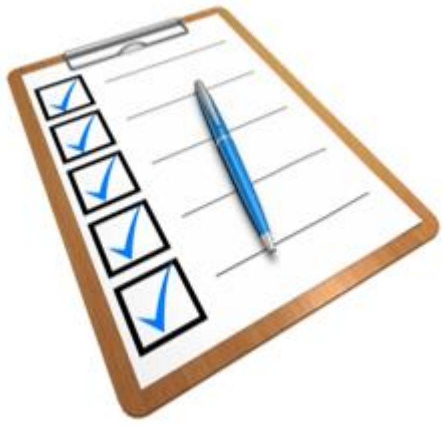

Those items are intended to be part of a **checklist** within the surveillance reporting guidelines.

For each item of the checklist, you will be asked to indicate your opinion: whether it is critical, optional or irrelevant to report that piece of information in order to correctly interpret surveillance outcomes.
There will also be a possibility to suggest items that have not been considered in our provisional checklist.

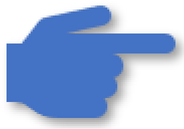

Please **download the complete checklist**: AHSURED_guidelines.pdf.
Reading it in advance will ease and speed up the reviewing process.

1. SURVEILLANCE SYSTEM - CONTEXT

The following items refer to the description of the context in which the surveillance system specifically operates.

For each of them, please indicate whether you believe is critical, optional or irrelevant to report to correctly interpret surveillance results. Feel free to add comments to your choices.

Item	Description	Relevance
**1.1 Hazard**	Hazard targeted by surveillance (a disease or another health-threat).<br>The surveillance activity may be multi-hazard. This is defined here as surveillance activities designed to target multiple hazards at the same time (parallel design), or the secondary use of data/samples collected as part of a surveillance activity designed for one specific hazard (mother component) to investigate the presence of additional hazards (child components). Should be mentioned if the surveillance of the hazard in question is part of a multi-hazard design.	□ Critical<br>□ Optional<br>□ Irrelevant
**1.2 Geographical area**	The geographical area, which the surveillance is designed to provide evidence about.	□ Critical<br>□ Optional<br>□ Irrelevant
**1.3 Susceptible population**	The specific animal population susceptible to the hazard in the geographical area of interest.	□ Critical<br>□ Optional<br>□ Irrelevant
**1.4 Historical evolution**	Describe the historical status of the hazard in the area and population in question.	□ Critical<br>□ Optional<br>□ Irrelevant

**1.5 Surveillance objective**	The objective of surveillance is a key characteristic which determines the most appropriate approach for the surveillance.<br>Prevalence estimation: This objective is appropriate if the target hazard is (thought to be) present in the territory to be covered, and an assessment of the prevalence and or changes in prevalence over time is needed.<br>Case detection: Case detection refers to the search for any animals affected, clinically or not, by the hazard under surveillance. This objective is appropriate if the hazard is present, regardless of whether the hazard occurrence is endemic, epidemic or sporadic, and action will be taken to control disease whenever it is detected.<br>Early detection: This objective is appropriate for a situation where the disease is currently absent in the country or region. It may be applied to surveillance for an unknown disease or a known disease considered to pose a non-negligible risk of incursion or emergence. It may also apply to the early stages of an outbreak.<br>Disease freedom: This objective is appropriate if the target hazard is thought to be absent in the territory to be covered and the aim is to demonstrate disease freedom for trade or non-trade purposes (e.g. improve public health, to decide when to stop an eradication programme and to eliminate production losses and control costs due to endemic disease). This may apply to diseases which a) have been historically absent, b) have been eradicated and official freedom has been confirmed or c) are thought to be eradicated, but official freedom has not yet been confirmed.<br>Surveillance systems may be used to provide information to address several surveillance objectives, but there is usually one that can be considered as primary. If other (secondary) surveillance objectives are relevant, they can be described for the components in question.	□ Critical<br>□ Optional<br>□ Irrelevant
**1.6 Surveillance purpose**	How is the information collected in this surveillance system being used to inform policy decision (e.g. to eradicate or manage the occurrence of disease or inform trade)?	□ Critical<br>□ Optional<br>□ Irrelevant
**1.7 Risk characteristics (level, aspect of risk)**	Risk characteristics associated with the hazard distribution and the susceptible population. Risk can be non-uniformly distributed at various level, such as population (particularly geographical and temporal risk factors), herd or animal. Risk characteristics should be described (whether they can be associated with a higher risk of hazard introduction, infection, detection, and/ or consequences).	□ Critical<br>□ Optional<br>□ Irrelevant

**1.8 Legal requirements, actions taken as a result of findings**	Describe the legal support underlying the surveillance, and whether it is a requirement or not to carry out surveillance. Describe also if there are any legal obligations to report, or other requirements (may be associated with quality assurance schemes, declarations for trade etc).<br>Describe what actions are taken in case of a positive result, if any.	□ Critical<br>□ Optional<br>□ Irrelevant
**1.9 Institutions involved, financing**	Mention institutions involved in coordination, field work, laboratory work, financing.<br>Describe the basis for financing.	□ Critical<br>□ Optional<br>□ Irrelevant

- Do you think that the item descriptions are clear enough? If not, please indicate what is unclear and/or how to improve one or more definitions.
- Do you think that this section is comprehensive or is there anything missing? Consider that missing item(s) might be mentioned further down in the checklist under a different section. If this is not the case, please feel free to indicate any additional item that you consider worth of being included under this section. Describe your suggested additional surveillance item(s), indicating also the relevance (optional or critical).

2. SURVEILLANCE COMPONENT - CHARACTERISTICS

The following item should be provided separately for each component within the system previously described

For each of them, please indicate whether you believe is critical, optional or irrelevant to report to correctly interpret surveillance results. Feel free to add comments to your choices.

Item	Description	Relevance
**2.1 Surveillance component objective**	Describe if different from main surveillance objective listed at 1.5<br>A surveillance system may for instance aim at demonstrating freedom (main objective) but one of its components (e.g. passive clinical surveillance) may aim at early detection (component-specific objective)	□ Critical<br>□ Optional<br>□ Irrelevant
**2.2 Target species**	Describe if different from 1.3	□ Critical<br>□ Optional<br>□ Irrelevant
**2.3 Target sector**	Describe if different from 1.3	□ Critical<br>□ Optional<br>□ Irrelevant
**2.4 Geographical area**	Describe if different/smaller than 1.2	□ Critical<br>□ Optional<br>□ Irrelevant
**2.5 Data collection point**	Where the units of interest can be reached, and therefore where samples will be collected.<br>This could be, e.g., at the source (farm, wild life habitat, etc), abattoir, coordination centre, artificial insemination centre, rendering plants, diagnostic laboratory, markets etc	□ Critical<br>□ Optional<br>□ Irrelevant

**2.6 Study type**	Describe the type of study (study types in RISKSUR framework listed below):<br>• Survey<br>• Continuous data collection<br>• Sentinel surveillance<br>• Participatory surveillance<br>• Indicator-based surveillance<br>• Syndromic surveillance<br>(For passive surveillance components, please refer to sections 4 and 5).	□ Critical<br>□ Optional<br>□ Irrelevant
**2.7 Type of disease indicator**	What outcome is measured for the unit of interest? E.g. is the surveillance aimed at antibody detection, pathogen detection, gross pathology, pathology diagnostic (microscopic) or indirect indicators etc?	□ Critical<br>□ Optional<br>□ Irrelevant
**2.8 Type of sample collected**	What is the type of material collected? Examples are, e.g. clinical reports, blood/serum/plasma, ear notches, tissue (biopsy), tissue (post mortem), milk, semen, urine, feces/ fecal swabs, other swab (not fecal), meat juice, environmental samples or feed.	□ Critical<br>□ Optional<br>□ Irrelevant

- Do you think that the item descriptions are clear enough? If not, please indicate what is unclear and/or how to improve one or more definitions.

- Do you think that this section is comprehensive or is there anything missing? Consider that missing item(s) might be mentioned further down in the checklist under a different section. If this is not the case, please feel free to indicate any additional item that you consider worth of being included under this section. Describe your suggested additional surveillance item(s), indicating also the relevance (optional or critical).

3. SURVEILLANCE COMPONENT - TARGET POPULATION

The following items refer to the target population of a specific surveillance component, which may be a subset of the whole population targeted by the surveillance system. They are expected to be provided separately for each component within the system previously described, if applicable.

For each of these items, please indicate whether you believe is critical, optional or irrelevant to report to correctly interpret surveillance results. Feel free to add comments to your choices.

Item	Description	Relevance
**3.1 Sectors missed**	Consider if there are other sectors/strata from the total susceptible population (defined in the surveillance scenario) that are not being covered by this particular component.	□ Critical<br>□ Optional<br>□ Irrelevant
**3.2 Target criteria**	Describe any selection criteria used to choose this particular target population (if the target population for this component is not the entire susceptible population defined for the surveillance system). For example; logistic/convenience; higher probability of infection; higher probability of showing clinical signs; feasibility of detection (the diagnostic tests available can only be used in animals above a certain age, or non-vaccinated animals); or higher severity of consequences in case of infection.	□ Critical<br>□ Optional<br>□ Irrelevant
**3.3 Percentage covered**	The percentage of the total susceptible population (defined in the surveillance scenario) covered by the target population defined for this specific component. This could be, for example, the percentage of the cattle population that is dairy for a component focused on dairy cows.	□ Critical<br>□ Optional<br>□ Irrelevant

- Do you think that the item descriptions are clear enough? If not, please indicate what is unclear and/or how to improve one or more definitions.
- Do you think that this section is comprehensive or is there anything missing? Consider that missing item(s) might be mentioned further down in the checklist under a different section. If this is not the case, please feel free to indicate any additional item that you consider worth of being included under this section. Describe your suggested additional surveillance item(s), indicating also the relevance (optional or critical).

4. Surveillance component - Disease Suspicion

The following items describe how a suspected case of the hazard of interest is defined and reported to the relevant authorities. This is relevant to passive surveillance components where the collection of surveillance data is observer-initiated. They are expected to be provided separately for each component within the system previously described, when applicable.

For each of these items, please indicate whether you believe is critical, optional or irrelevant to report to correctly interpret surveillance results. Feel free to add comments to your choices.

Item	Description	Relevance
**4.1 Criteria for identification of suspicions**	Outline the definition or criteria used to identify a suspect case.	□ Critical<br>□ Optional<br>□ Irrelevant
**4.2 Obligations on suspicions**	Describe requirements in place for reporting of a suspect case.	□ Critical<br>□ Optional<br>□ Irrelevant
**4.3 Notification procedures**	Describe the procedures for reporting a suspect case, i.e. the steps involved and the methods employed, including how the notification is sent to the authorities.	□ Critical<br>□ Optional<br>□ Irrelevant
**4.4 Actions upon suspicions**	Describe the procedures following the reporting of a suspect case to the authorities, for example at what stage would/could restrictions are applied to premises, or follow up investigations carried out.	□ Critical<br>□ Optional<br>□ Irrelevant

- Do you think that the item descriptions are clear enough? If not, please indicate what is unclear and/or how to improve one or more definitions.
- Do you think that this section is comprehensive or is there anything missing? Consider that missing item(s) might be mentioned further down in the checklist under a different section. If this is not the case, please feel free to indicate any additional item that you consider worth of being included under this section. Describe your suggested additional surveillance item(s), indicating also the relevance (optional or critical).

5. Surveillance component - Disease Suspicion

The following item describes how participation in surveillance and control activities is encouraged. This is relevant to enhanced-passive surveillance components, where the collection of surveillance data is observer-initiated after incentives.
It is expected to be provided separately for each component within the system previously described, when applicable.

Please indicate whether you believe this item is critical, optional or irrelevant to report to correctly interpret surveillance results. Feel free to add comments to your choice.

Item	Description	Relevance
**5.1 Enhancements in place**	Describe any enhancements in place to encourage participation in surveillance and control activities, such as awareness campaigns and monetary or other incentives to reporting. Examples are:<br>• payments or other financial rewards for notifications<br>• training to increase awareness and recognition of clinical signs<br>• awareness campaigns to improve recognition of disease and awareness of reporting obligations/procedures.<br>• payment of financial compensation for the losses the farmer may incur after a confirmation of notifiable disease<br>• provision of alternative routes of reporting such as a phone hotline or notification by SMS<br>• payment of the testing cost or some other form of mutual agreement such as farmer receiving advice in return<br>• mandatory/legal obligation to carry out diagnosis of exclusion	□ Critical<br>□ Optional<br>□ Irrelevant

- Do you think that the item description is clear enough? If not, please indicate what is unclear and/or how to improve the definition.

- Do you think that this section is comprehensive or is there anything missing? Consider that missing item(s) might be mentioned further down in the checklist under a different section. If this is not the case, please feel free to indicate any additional item that you consider worth of being included under this section. Describe your suggested additional surveillance item(s), indicating also the relevance (optional or critical).

6. Surveillance component – Testing protocol

The following items describe how units have been tested to obtain information about the hazard. If passive surveillance is being adopted, the testing protocol should be detailed for the confirmation of suspicions (definition of suspicions was addressed in section 4).
They are expected to be provided separately for each component within the system previously described, if applicable.

For each of these items, please indicate whether you believe is critical, optional or irrelevant to report to correctly interpret surveillance results. Feel free to add comments to your choices.

Item	Description	Relevance
**6.1 Pooling**	If pooling has been done, please describe how and where (field, lab etc).	□ Critical<br>□ Optional<br>□ Irrelevant
**6.2 Screening/first test**	Describe for the screening test (or only test), any thresholds for considering an animal as positive.	□ Critical<br>□ Optional<br>□ Irrelevant
**6.3 Confirmatory/ second test**	For confirmatory tests (in cases where screening is used), or the second test (when using parallel tests), describe the test to be used, and any thresholds for confirming animals as positive.	□ Critical<br>□ Optional<br>□ Irrelevant
**6.4 Any other testing protocol details**	Any further details needed to make sure the entire process is well described.	□ Critical<br>□ Optional<br>□ Irrelevant

- Do you think that the item descriptions are clear enough? If not, please indicate what is unclear and/or how to improve one or more definitions.
- Do you think that this section is comprehensive or is there anything missing? Consider that missing item(s) might be mentioned further down in the checklist under a different section. If this is not the case, please feel free to indicate any additional item that you consider worth of being included under this section. Describe your suggested additional surveillance item(s), indicating also the relevance (optional or critical).

7. SURVEILLANCE COMPONENT – STUDY DESIGN

The following items describe how the study population was selected. Was it a census (testing all animals in the target population) or was the population subjected to some type of selective sampling strategy? Even if a census was performed, one may still want to consider some information in this section, such as the number of units in the population, as they are important for data collection. Moreover, if the target units are clustered (for instance animals clustered in farms), and a census at the top level will be performed (census of herds for instance), sampling within clusters (animals within farms) may still have been designed.

Such items are expected to be provided separately for each component within the system previously described, if applicable.

For each of these items, please indicate whether you believe is critical, optional or irrelevant to report to correctly interpret surveillance results. Feel free to add comments to your choices.

Item	Description	Relevance
**7.1 Selection of units: census or sampling**	Describe if a census has been conducted, or if sampling has been applied. Describe the sampling frame used to select the units of interest.	□ Critical<br>□ Optional<br>□ Irrelevant
**7.2 Target unit level (unit of interest)**	Describe the target unit, i.e. the level of the population for which conclusions are to be drawn (for instance animal or herds).	□ Critical<br>□ Optional<br>□ Irrelevant
**7.3 Sampling unit - individual or group**	Describe the sampling units, i.e. the units that have actually been sampled (individuals; multiple group sample (collective/pooled samples which represent multiple animals, but not the entire target unit); one sample per group (collective/pooled samples which represent the entire group referred to in your target unit).	□ Critical<br>□ Optional<br>□ Irrelevant
**7.4 Sampling design**	Describe the sampling design, e.g. one- or two-stage.	□ Critical<br>□ Optional<br>□ Irrelevant

**7.5 Number of units in the target population**	Describe what is known about the size of the target population.	□ Critical<br>□ Optional<br>□ Irrelevant
**7.6 Sensitivity of the testing protocol**	Describe what is known about the sensitivity of the testing protocol, incl. if tests are applied in series or parallel.	□ Critical<br>□ Optional<br>□ Irrelevant
**7.7 Specificity of the testing protocol**	Describe what is known about the specificity of the testing protocol, incl. if tests are applied in series or parallel.	□ Critical<br>□ Optional<br>□ Irrelevant

- Do you think that the item descriptions are clear enough? If not, please indicate what is unclear and/or how to improve one or more definitions.

- Do you think that this section is comprehensive or is there anything missing? Consider that missing item(s) might be mentioned further down in the checklist under a different section. If this is not the case, please feel free to indicate any additional item that you consider worth of being included under this section. Describe your suggested additional surveillance item(s), indicating also the relevance (optional or critical).

8. SURVEILLANCE COMPONENT – SAMPLING STRATEGY

The following items describe how units have been planned to be sampled to obtain information about the hazard.
They are expected to be provided separately for each component within the system previously described, if applicable.

For each of these items, please indicate whether you believe is critical, optional or irrelevant to report to correctly interpret surveillance results. Feel free to add comments to your choices.

Item	Description	Relevance
**8.1 Sampling at the primary sampling unit (PSU) level**	Information used to calculate sample size at the PS level, e.g. the number of PSU in the population (see section 7 also), design prevalence, desired confidence, desired power, sensitivity, specificity.	□ Critical<br>□ Optional<br>□ Irrelevant
**8.2 Sampling at the secondary sampling unit (SSU) level**	Information used to calculate sample size at the SS level, e.g. the number of SSU in the population (see section 7 also), design prevalence, desired confidence, desired power, sensitivity, specificity.	□ Critical<br>□ Optional<br>□ Irrelevant
**8.3 Selection criteria within the population**	See 3.2 Target criteria (= selection criteria used to choose this particular target population when the target population for this component is not the entire susceptible population defined for the surveillance system).	□ Critical<br>□ Optional<br>□ Irrelevant
**8.4 Risk-based allocation**	Describe the factors defining any risk strata. For each risk stratum defined, the following information should be reported:<br>a) risk characteristics: higher probability of infection, detection, consequences etc (revisit section 1.7)<br>b) percentage of the population that the stratum constitutes<br>d) relative risks	□ Critical<br>□ Optional<br>□ Irrelevant
**8.5 Sample size**	The following details should be recorded:<br>a) sample size calculated at PSU level<br>b) sample size calculated at SSU level<br>c) planned distribution of samples over the year	□ Critical<br>□ Optional<br>□ Irrelevant

**8.6 Sample allocation at the primary and secondary levels**	Describe the strategy for allocating samples. 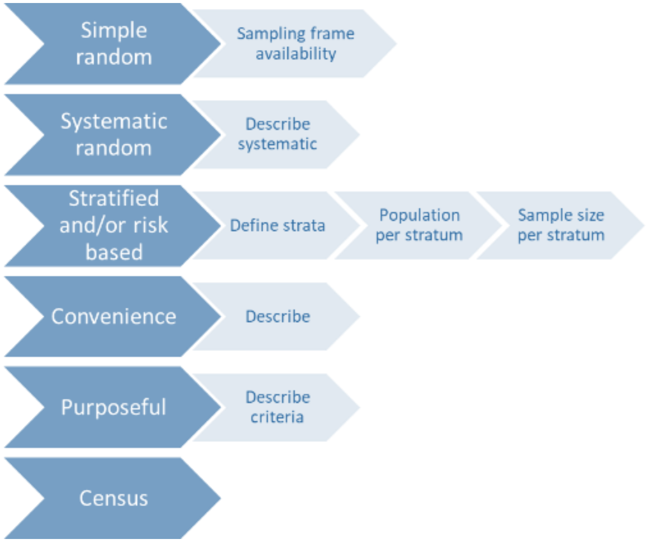	□ Critical<br>□ Optional<br>□ Irrelevant
**8.7 Sample collection timeline**	Describe how the sampling has been distributed across the study period (sampling plan).	□ Critical<br>□ Optional<br>□ Irrelevant

- Do you think that the item descriptions are clear enough? If not, please indicate what is unclear and/or how to improve one or more definitions.

- Do you think that this section is comprehensive or is there anything missing? Consider that missing item(s) might be mentioned further down in the checklist under a different section. If this is not the case, please feel free to indicate any additional item that you consider worth of being included under this section. Describe your suggested additional surveillance item(s), indicating also the relevance (optional or critical).

9. SURVEILLANCE COMPONENT – DATA GENERATION PROCESS

The following items describe the specific process of collecting the samples (or any other information) from the source.
They are expected to be provided separately for each component within the system previously described, if applicable.

For each of these items, please indicate whether you believe is critical, optional or irrelevant to report to correctly interpret surveillance results. Feel free to add comments to your choices.

Item	Description	Relevance
**9.1 Who collects the samples?**	Who are the agents who collect samples/information?<br>Examples of agents who may collect samples are:<br>▪ non-specialised actors (farmers, public, hunters, etc);<br>▪ technicians;<br>▪ veterinarians.	□ Critical<br>□ Optional<br>□ Irrelevant
**9.2 When/how often are samples collected?**	For surveillance where timeliness is important (early detection, case finding), describe how often samples have been collected across the study period.	□ Critical<br>□ Optional<br>□ Irrelevant

➢ Do you think that the item descriptions are clear enough? If not, please indicate what is unclear and/or how to improve one or more definitions.

➢ Do you think that this section is comprehensive or is there anything missing? Consider that missing item(s) might be mentioned further down in the checklist under a different section. If this is not the case, please feel free to indicate any additional item that you consider worth of being included under this section. Describe your suggested additional surveillance item(s), indicating also the relevance (optional or critical).

10. Surveillance component – Transfer means

The following item describe how data/samples are transferred from the point of collection to the point of analyses. The point of analysis for samples is typically the laboratory, and the point of analysis for data is typically an epidemiologist.
It is expected to be provided separately for each component within the system previously described, if applicable.

Please indicate whether you believe that this item is critical, optional or irrelevant to report to correctly interpret surveillance results. Feel free to add comments to your choice.

Item	Description	Relevance
**10.1 When/how often are samples transferred**	For surveillance where timeliness is important (early detection, case finding), describe how often samples have been transferred across the study period.	□ Critical<br>□ Optional<br>□ Irrelevant

- Do you think that the item description is clear enough? If not, please indicate what is unclear and/or how to improve the definition.
- Do you think that this section is comprehensive or is there anything missing? Consider that missing item(s) might be mentioned further down in the checklist under a different section. If this is not the case, please feel free to indicate any additional item that you consider worth of being included under this section. Describe your suggested additional surveillance item(s), indicating also the relevance (optional or critical).

11. SURVEILLANCE COMPONENT – DATA TRANSLATION PROCESS

The following items describe how the raw data (biological samples, health indicators, observations etc) is translated into surveillance information. The type of samples to be collected and testing to be carried out has been determined in section 6 (testing protocol). Here details should be recorded regarding the management and logistical aspects of the analysis of the raw data/samples in order to turn it into useful surveillance data.
They are expected to be provided separately for each component within the system previously described, if applicable.

For each of these items, please indicate whether you believe is critical, optional or irrelevant to report to correctly interpret surveillance results. Feel free to add comments to your choices.

Item	Description	Relevance
**11.1 Who has performed the analyses?**	Who has been responsible for the analysis of the raw data/samples, official laboratory status, accreditation etc	□ Critical<br>□ Optional<br>□ Irrelevant
**11.2 When/how often are samples analysed**	For surveillance where timeliness is important (early detection, case finding), describe how often samples will be analysed across the study period.<br>This could for example be; as soon as received (immediate/real-time).;according to a fixed schedule eg weekly, monthly; or, in batches after a certain number of samples is reached.	□ Critical<br>□ Optional<br>□ Irrelevant

- Do you think that the item descriptions are clear enough? If not, please indicate what is unclear and/or how to improve one or more definitions.

- Do you think that this section is comprehensive or is there anything missing? Consider that missing item(s) might be mentioned further down in the checklist under a different section. If this is not the case, please feel free to indicate any additional item that you consider worth of being included under this section. Describe your suggested additional surveillance item(s), indicating also the relevance (optional or critical).

12. Surveillance component – Epidmiological Analyses

Once samples are analysed by the laboratories or interpreted by personnel, it is expected that the results of those analyses will be reviewed by epidemiologists and other involved in the study design for this surveillance component.
This item is expected to be provided separately for each component within the system previously described, if applicable.

Please indicate whether you believe that this item is critical, optional or irrelevant to report to correctly interpret surveillance results. Feel free to add comments to your choice.

Item	Description	Relevance
**12.1 When/how often are data analysed**	For surveillance where timeliness is important (early detection, case finding), describe how often data have been looked upon, analysed and interpreted during the study period.	□ Critical<br>□ Optional<br>□ Irrelevant

- Do you think that the item description is clear enough? If not, please indicate what is unclear and/or how to improve the definition.
- Do you think that this section is comprehensive or is there anything missing? Consider that missing item(s) might be mentioned further down in the checklist under a different section. If this is not the case, please feel free to indicate any additional item that you consider worth of being included under this section. Describe your suggested additional surveillance item(s), indicating also the relevance (optional or critical).

13. SURVEILLANCE COMPONENT – RESULTS

The following items represent the outcome of the surveillance activities for the described component.
They are expected to be provided separately for each component within the system previously described.

For each of these items, please indicate whether you believe is critical, optional or irrelevant to report to correctly interpret surveillance results. Feel free to add comments to your choices.

Item	Description	Relevance
**13.1 Number of epidemiological units investigated (per stratum)**	Report the number of epidemiological units investigated, by relevant strata and overall.	□ Critical<br>□ Optional<br>□ Irrelevant
**13.2 Test results (per stratum)**	Report the outcome of the testing, by relevant strata and overall. Report sampling efforts results spatially and temporally.	□ Critical<br>□ Optional<br>□ Irrelevant
**13.3 Surveillance outcomes (objective dependent)**	Provide an assessment of the outcome of surveillance objective in relation to its objective.<br>For prevalence estimation, this means prevalence with an accompanying confidence interval.<br>For case finding, the outcome of interest is incidence or the detection fraction.<br>For freedom studies, the outcome is probability of freedom, with accompanying design prevalence and confidence level (should be reported at several confidence levels/design prevalences).<br>For surveillance aimed at early detection, where no cases have been found, report intensity and relation to risk-based sampling activities.	□ Critical<br>□ Optional<br>□ Irrelevant
**13.4 Findings in relation to historical knowledge, trend**	Provide a narrative and/or graphical description of the evolution of surveillance outcomes over time. If a trend analyses is carried out, describe method.	□ Critical<br>□ Optional<br>□ Irrelevant

- Do you think that the item descriptions are clear enough? If not, please indicate what is unclear and/or how to improve one or more definitions.

- Do you think that this section is comprehensive or is there anything missing? Consider that missing item(s) might be mentioned further down in the checklist under a different section. If this is not the case, please feel free to indicate any additional item that you consider worth of being included under this section. Describe your suggested additional surveillance item(s), indicating also the relevance (optional or critical).

14. Surveillance component – Interpretation

Please indicate whether you believe that this item is critical, optional or irrelevant to report to correctly interpret surveillance results. Feel free to add comments to your choice.

Item	Description	Relevance
**14.1 Interpretation**	Summarise what evidence the surveillance activities provide about the status of the population, taking into account issues about coverage, representativeness/bias as well as generalisability. If relevant, describe any future plans to continue activities.	□ Critical<br>□ Optional<br>□ Irrelevant

- Do you think that the item description is clear enough? If not, please indicate what is unclear and/or how to improve the definition.

- Do you think that this section is comprehensive or is there anything missing? Consider that missing item(s) might be mentioned further down in the checklist under a different section. If this is not the case, please feel free to indicate any additional item that you consider worth of being included under this section. Describe your suggested additional surveillance item(s), indicating also the relevance (optional or critical)

15. SURVEILLANCE COMPONENT – REFERENCES

Please indicate whether you believe that this item is critical, optional or irrelevant to report to correctly interpret surveillance results. Feel free to add comments to your choice.

Item	Description	Relevance
**14.1 References**	Provide references to support, e.g. methods and historical evidence.	□ Critical<br>□ Optional<br>□ Irrelevant

- Do you think that the item description is clear enough? If not, please indicate what is unclear and/or how to improve the definition.

- Do you think that this section is comprehensive or is there anything missing? Consider that missing item(s) might be mentioned further down in the checklist under a different section. If this is not the case, please feel free to indicate any additional item that you consider worth of being included under this section. Describe your suggested additional surveillance item(s), indicating also the relevance (optional or critical)
-

ABOUT YOU...

As final step, we would like to know more about your professional background in relation to animal health surveillance.

1. Do the answers provided reflect your own opinion or a collective opinion from a group of peers?
   - ☐ My personal opinion
   - ☐ The opinion of a group I'm representing

2. What is your current involvement in animal health surveillance? Check as many as relevant.
   - ☐ Design of surveillance activities
   - ☐ Evaluation of surveillance performance
   - ☐ Reporting surveillance results at national/international level
   - ☐ Risk assessment
   - ☐ Risk management
   - ☐ Implementation of surveillance activities
   - ☐ Secondary use of data (e.g. for research purposes)
   - ☐ Other, please specify ______________________________

3. How long have you been working with animal health surveillance (design, implementation, evaluation, use of result for risk assessment or risk management, etc.)?
   If the group of people answering the survey had different levels of experience, indicate the longest experience within the group.Design of surveillance activities
   - ☐ Less than 5 years
   - ☐ Between 5 and 10 years
   - ☐ More than 10 years

4. What sector do you currently work for?
   - ☐ Public health
   - ☐ Ministry/Government
   - ☐ Academia
   - ☐ Private company
   - ☐ Other, please specify ____________________________________

5. From what areas of the world do you mostly have work experience?
If the group of people answering the survey had work experience from different areas, indicate the most represented one.
- ☐ Africa
- ☐ Asia
- ☐ Europe
- ☐ Oceania
- ☐ North America
- ☐ South America

6. We are forming a **core group of experts** to further validate the development of the guidelines. The expert group will be used to seek consensus on how to deal with items where there is significant divergence in opinion, to provide further input on item descriptions and/or gather real-world examples of well reported items.
Would you be interested in being part in the core group and in the further development of guidelines for reporting animal health surveillance?
Experts in the core group are expected to participate in up to two webinars where the guidelines will be consolidated. Their contribution will be acknowledged in the publication of the guidelines.
- ☐ No, thank you
- ☐ Yes, definitively!

7. In case you answered “yes” to the previous question, please provide an email address where we can contact you (individual respondent) or your group (multiple respondents) in the future.
____
