# Supplementary material for: Development of Reporting Guidelines for Animal Health Surveillance—AHSURED
Source: Front Vet Sci. 2019 Nov 27;6:426. doi: 10.3389/fvets.2019.00426 (PMC6890601; doi:10.3389/fvets.2019.00426)
Supplement: Supplementary Material 3 — Results of the web survey. [file Data_Sheet_3.pdf]

Results of the web survey

Number of respondents that judged each item critical, optional or irrelevant.

1. Surveillance System - Context

	Critical	Optional	Irrelevant	missing
1.1 Hazard	32	1	0	0
1.2 Geographical area	31	2	0	0
1.3 Susceptible population	26	6	0	1
1.4 Historical evolution	11	20	1	1
1.5 Surveillance objective	30	2	0	1
1.6 Surveillance purpose	23	9	0	1
1.7 Risk characteristics	21	8	1	3
1.8 Legal requirements	13	17	1	2
1.9 Institution involved, financing	15	14	3	1

2. Surveillance Component - Characteristics

	Critical	Optional	Irrelevant	missing
2.1 Surveillance component objective	28	4	0	1
2.2 Target species	30	3	0	0
2.3 Target sector	23	9	0	1
2.4 Geographical area	28	5	0	0
2.5 Data collection point	29	3	0	1
2.6 Study type	28	4	0	1
2.7 Type of disease indicator	31	1	0	1
2.8 Type of sample collected	30	2	0	1

3. Surveillance Component - Target Population

	Critical	Optional	Irrelevant	missing
3.1 Sector missed	22	10	1	0
3.2 Target criteria	27	6	0	0
3.3 Percentage covered	23	8	2	0

4. Surveillance Component - Disease Suspicion

	Critical	Optional	Irrelevant	missing
4.1 Criteria for identification of suspicions	32	1	0	0
4.2 Obligations on suspicions	30	2	1	0
4.3 Notification procedures	26	5	2	0
4.4 Actions upon suspicions	26	6	1	0

5. Surveillance Component - Enhancements

	Critical	Optional	Irrelevant	missing
5.1 Enhancements	16	16	0 0	1

6. Surveillance Component – Testing Protocol

	Critical	Optional	Irrelevant	missing
6.1 Pooling	27	6	0	0
6.2 Screening/first test	31	2	0	0
6.3 Confirmatory/ second test	30	3	0	0
6.4 Any other testing protocol details	14	16	1	2

7. Surveillance Component – Study Design

	Critical	Optional	Irrelevant	missing
7.1 Selection of units: census or sampling	31	2	0	0
7.2 Target unit level (unit of interest)	32	1	0	0
7.3 Sampling unit - individual or group	31	2	0	0
7.4 Sampling design	30	2	1	0
7.5 Number of units in the target population	26	7	0	0
7.6 Sensitivity of the testing protocol	31	2	0	0
7.7 Specificity of the testing protocol	31	2	0	0

8. Surveillance Component – Sampling Strategy

	Critical	Optional	Irrelevant	missing
8.1 Sampling at the primary sampling unit (PSU) level	31	2	0	0
8.2 Sampling at the secondary sampling unit (SSU) level	26	7	0	0
8.3 Selection criteria within the population	28	5	0	0
8.4 Risk-based allocation	28	5	0	0
8.5 Sample size	30	2	0	1
8.6 Sample allocation at the primary and secondary levels	24	9	0	0
8.7 Sample collection timeline	23	10	0	0

9. Surveillance Component – Data Generation Process

	Critical	Optional	Irrelevant	missing
9.1 Who collects the samples?	14	19	0	0
9.2 When/how often are samples collected?	27	6	0	0

10. Surveillance Component – Transfer Means

	Critical	Optional	Irrelevant	missing
10.1 When/how often are samples transferred	14	15	2	2

11. Surveillance Component – Data Translation Process

	Critical	Optional	Irrelevant	missing
11.1 Who has performed the analyses?	16	15	2	0
11.2 When/how often are samples analysed	21	12	0	0

12. Surveillance Component – Epidmiological Analyses

	Critical	Optional	Irrelevant	missing
12.1 When/how often are data analysed	16	16	1	0

13. Surveillance Component – Results

	Critical	Optional	Irrelevant	missing
13.1 Number of epidemiological units investigated	28	5	0	0
13.2 Test results	26	7	0	0
13.3 Surveillance outcomes (objective dependent)	29	4	0	0
13.4 Findings in relation to historical knowledge, trend	15	18	0	0

14. Surveillance Component – Interpretation

	Critical	Optional	Irrelevant	missing
14.1 Surveillance interpretation	31	2	0	0

15. SURVEILLANCE COMPONENT – REFERENCES

	Critical	Optional	Irrelevant	missing
15.1 References	16	15	2	0
